# Synthesis of Three Rimantadine Schiff Bases and Their Biological Effects on Serum Albumin

**Published:** 2014

**Authors:** Bing-Mi Liu, Ping Ma, Xin Wang, Yu-Mei Kong, Li-Ping Zhang, Bin Liu

**Affiliations:** a *College of Pharmacy, Liaoning University, Shenyang 110036, P. R. China.*; b *Department of Clinical Pharmacology, General Hospital of the Rocket Force, Beijing 100088, P.R.China.*

**Keywords:** Rimantadine schiff base (RSB), Bovine serum albumin (BSA), Interaction, Fluorescence

## Abstract

Three new rimantadine Schiff bases (RSBs) were prepared, and then the interaction of RSBs with bovine serum albumin (BSA) was investigated using fluorescence, synchronous fluorescence, UV-vis absorption spectroscopy under physiological conditions. The results showed that the three RSBs effectively quenched the intrinsic fluorescence of BSA via static quenching. Binding constant (*K*_a_), number of binding sites (*n*), and the binding distance (*r*) between three RSBs and BSA were calculated by Stern-Volmer equation and Förster’s theory in this study. According to the results of displacement experiments of site probes, it was considered that the binding sites were located in hydrophobic cavities in sub-domains IIA of BSA. What is more, synchronous fluorescence studies indicated that the hydrophobicity around tryptophan residues was increased with the addition of rimantadine-o-vanillin (ROV) and rimantadine-4-methoxy-salicylaldehyde (RMS), while there was no apparent change with the addition of rimantadine-salicylaldehyde (RS).

## Introduction

Schiff bases are a category of compounds containing C=N group. It is reported that many Schiff bases show a variety of interesting biological actions, including antibacterial, antifungal, anticancer and antiviral actions (1, 2). So, in recent years, some researches have been concentrated on them (3-5).

Rimantadine hydrochloride (*α*-methyl-1-adamantane methylamine hydrochloride), which exhibits equal efficacy and fewer adverse reactions than amantadine hydrochloride, has been clinically used for therapy of infections caused by a broad range of RNA-containing viruses, in particular the influenza A virus (6,7). It has also been reported that rimantadine hydrochloride has some effects on Parkinson’s disease. Salicylaldehyde and its derivatives are a kind of important chemical-industrial raw material and organic synthesis midbody (8). Also, they are often used in medicine chemical engineering (9) because of their biological activities, such as anti-inflammatory, antibacterial and antiviral activities (10, 11).

**Figure 1:**
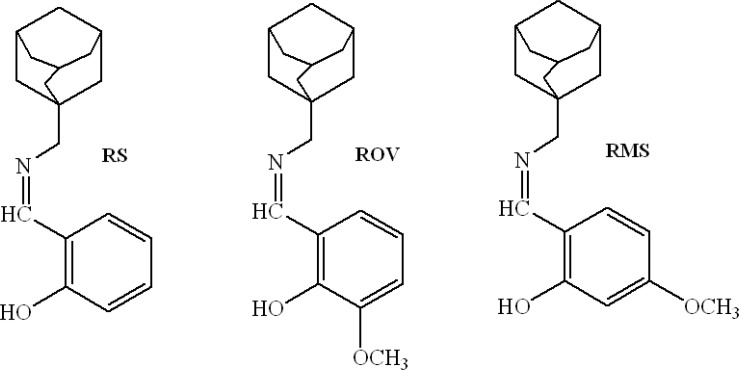
Molecular structures of rimantadine-salicylaldehyde (RS), rimantadine-o-vanillin (ROV) and rimantadine-4-methoxy- salicylaldehyde (RMS) Schiff bases

In recent years, the new pattern influenzal characteristic is often amphibianous. That is, the influenza is caused by viruses and germs at the same time (12, 13). Therefore, it is necessary to design the difunctional drugs, which have antiviral and antibacterial actions simultaneously (14). In this work, three new rimantadine Schiff bases (RSBs), rimantadine-salicylaldehyde (RS), rimantadine-o-vanillin (ROV) and rimantadine-4-methoxy-salicylaldehyde (RMS), were synthesized which would display the better biologic activities. The molecular structures of three RSBs are shown in Figure 1.

Protein plays an important role in life processes. Serum albumins are the most abundant proteins in the plasma and have many physiological functions, the most outstanding property of which is their ability to bind reversibly a large variety of ligands, such as cystein, glutathione, Schiff base ligands (15, 16) and so on. The binding ability of drug-albumin in blood stream may have a significant impact on distribution, free concentration, and metabolism of drug. Thus, it is important and necessary to study the interaction of drug with serum albumins at molecular level.

Spectroscopic method has been widely applied in investigating drug binding with albumin because of its accuracy, sensitivity, rapidity and convenience of usage (17). Hence, in this study, the interaction of RSBs (RS, ROV, RMS) with BSA was investigated via UV-vis absorption and fluorescence spectroscopy. Besides, the binding sites of RSBs to BSA molecules were also explored by site probes (ketoprofen and ibuprofen) (18). 

## Experimental

Rimantadine hydrochloride was obtained from Jinan Dachpharm Development Co., *Ltd*., China. Salicylaldehyde was purchased from Sinopharm Chemical Reagent Co., *Ltd*., China. The o-vanillin and 4- methoxy-salicylaldehyde were obtained from Tianjing Tianhe Chemical Reagent Co., *Ltd*. Bovine serum albumin (BSA, purity > 99.0 %) was purchased from Beijing Abxing Biological Technology Company. The BSA solution of 2.00 × 10^-5^ mol/L was prepared by dissolving 1.340 g BSA in 1 L buffer and kept in the dark at 4 °C. Buffer (pH 7.4) consisted of Tris (0.2 mol/L) and HCl (0.1 mol/L), and the ion strength was maintained by adding 0.05 mol/L NaCl. All other chemicals were of analytical reagent grade.

The structures of RSBs were analysed by an AV-600 NMR instrument (Bruker, Germany) and a Fourier transform infrared spectrophotometer (Spectrum 100, PerkinElmer Company, USA). Fluorescence emission spectra and synchronous spectra were collected on an F-7000 spetrofluorophotometer (Hitachi, Japan). The absorption spectra were recorded on a Cary 50 UV-vis spectrophotometer (Varian, USA). 

*Syntheses of* *r* *imantadine* *-* *s* *alicylaldehyde* *(* *RS* *)* *,* *r* *imantadine* *-* *o-* *v* *anillin* *(ROV)* *and* *r* *imantadine* *-* *4-* *m* *ethoxy-* *s* *alicylaldehyde* *(RMS)*

4.747 g (0.022 mol) rimantadine hydrochloride and 1.234 g (0.022 mol) KOH were added to 50 mL ethanol and then stirred for 2 h at room temperature. The precipitate was filtered off, and then the solution was transferred to a flask. To this solution, 10 mL salicylaldehyde-ethanol solution (containing 0.02 mol salicylaldehyde) was added drop-wise and stirred at 80 °C for 2 h. After 2 h the reaction mixture was concentrated until RS (yellow precipitate) appeared on the bottom. Naturally cooling to room temperature, the solid mass formed was filtered and washed with ethanol three times and dried at 60 °C. 

According to the method above, ROV and RMS were also synthesized. The related data as follows:

RS, m.p. 88–89 °C; 1H NMR (CDCl_3_) δ: 1.18 (d, 3H, CH_3_), 1.56 (s, 6H, CH_2_), 1.67 (dd, J = 12.0, 42.6 Hz, 6H, CH_2_), 1.99 (s, 3H, CH), 2.83 (q, 1H, N-CH), 6.86 (t, J = 7.8 Hz, 1H, Ph), 6.95 (d, J = 8.4 Hz, 1H, Ph), 7.24 (dd, J = 1.2, 7.2 Hz, 1H, Ph), 7.30 (m, 1H, Ph), 8.26 (s, 1H, CH=N), 13.91 (s,1H, Ar-OH).

ROV, m.p. 86–88 °C; 1H NMR (CDCl_3_) δ: 1.18 (d, 3H, CH_3_), 1.56 (s, 6H, CH_2_), 1.66 (dd, J = 12.0, 41.4 Hz, 6H, CH_2_), 1.97 (s, 3H, CH), 2.87 (q, 1H, N-CH), 3.90 (s, 3H, OCH_3_), 6.76 (t, J = 7.8 Hz, 1H, Ph), 6.86 (dd, J = 1.2, 7.2 Hz, 1H, Ph), 6.90 (dd, J = 1.2, 7.8 Hz, 1H, Ph), 8.23 (s, 1H, CH=N), 14.59 (s,1H, Ar-OH).

RMS, m.p. 103–104 °C; 1H NMR (CDCl_3_) δ: 1.19 (d, 3H, CH_3_), 1.55(s, 6H, CH_2_), 1.67 (dd, J = 12.0, 42.6 Hz, 6H, CH_2_), 1.99 (s, 3H, CH), 2.84 (q, 1H, N-CH), 3.80 (s, 3H, OCH_3_), 6.33 (dd, J = 2.4, 8.4 Hz, 1H, Ph), 6.38 (d, J = 3.0 Hz, 1H, Ph), 7.06 (d, J = 8.4 Hz, 1H, Ph), 8.02 (s, 1H, CH=N), 14.40 (s,1H, Ar-OH).

At the same time, their infrared spectra were also determined by using a Fourier transform infrared spectrophotometer. The corresponding results were offered in Table 1. 

*Spectroscopic measurements*

The absorption spectra of the three RSBs were collected. At the same time, the fluorescence spectra of BSA with and without RSBs were performed at 290 K in the range of 200-500 nm upon excitation at 280 nm. The widths of both the entrance and exit slit were set to 5 nm. The concentration of BSA firstly was fixed at 1.00 × l0^-5^ mol/L, and then titrated with different amount of RSBs’ stock solution (2.50   10^-^^3 ^mol/L), which was prepared by dissolving the appropriate amount of RSBs with alcohol, and then diluting with Tris-HCl-NaCl buffer in 100 mL volumetric flask.

For every addition, the mixture solution must be shaken and allowed to stand for 10 min. The synchronous fluorescence spectra were recorded from 200 to 500 nm at λ = 15 and λ = 60 nm, respectively. Appropriate blanks corresponding to the buffer were subtracted to correct the fluorescence or absorption background.

## Results and Discussion

*Infrared* *s* *pectra of* *RS* *,* *ROV* *and* *RMS*

Some physical data of three new RSBs and the most important IR peaks of the ligand are reported in Table 1. It can be seen that the (C=O) of salicylaldehyde at 1663 cm^–1^ disappears and the (N－H) at 2902 cm^–1^ of rimantadine also disappears, then a new peak appears at 1630 cm^–1^, which could be attributed to C=N vibrations and support the formation of the RS. Similarly, new peaks observed at 1628 cm^–1^ for ROV and at 1622 cm^–1^ for RMS indicate the formation of the C=N bond. 

**Table 1:** Physical and Infrared spectral data of RS, ROV and RMS

**System**	**Colour**	**Melting ** **Point (ºC)**	**(N－H) (cm**^-1^**)**	** (C=O)** ** (cm** ^-1^ **)**	** (C=N)** **(cm**^-1^**)**
Rimantadine			2902		
Salicylaldehyde				1663	
RS	yellow	88-89			1630
o-Vanillin				1639	
ROV	yellow	86-88			1628
4-Methoxy- Salicylaldehyde				1659	
RMS	pale yellow	103-104			1622

*Fluorescence* *s* *pectra of BSA* *s* *olutions with* *RS* *,* *ROV* *and RMS*

**Figure 2:**
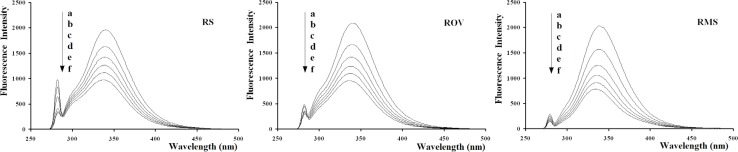
Fluorescence quenching spectra of BSA in the presence of RSBs (RS, ROV, RMS). ([RSBs](a→f) = 0.00, 0.50, 1.00, 1.50, 2.00, 2.50   10^-5^ mol/L, [BSA] = 1.00   10^-5^ mol/L).

Figure 2 shows the emission spectra of BSA in the absence and presence of RSBs (RS, ROV, RMS). As can be seen from Figure 2, BSA had strong fluorescence emission with a peak at 340 nm on excitation at 280 nm. Furthermore, the addition of RSBs led to a concentration-dependent quenching of BSA intrinsic fluorescence intensity and the maximum emission wavelengths were slightly shifted from 340 nm to 337 nm for RS and ROV, 340 nm to 336 nm for RMS. These results indicate that the binding of RSBs to BSA occurs and the microenvironment around chromophore of BSA is changed upon addition of RSBs (RS, ROV, RMS).

To interpret the data from fluorescence quenching studies, it is important to understand what kinds of interaction take place between the fluorophore (BSA) and the quencher (RSBs). If it is assumed that the fluorescence quenching of BSA induced by RSBs are dynamic quenching process, fluorescence quenching is described by the Stern-Volmer equation:

 Equation (1) $$F_{0}/F=1+K_{q}t_{0}[Q]=1+\mathrm{K\_SV}[Q]$$

The *F*_0_ and *F* are the fluorescence intensities in the absence and presence of quencher, respectively. *K*_q_ is the quenching rate constant, *τ*_0_ the average lifetime of the biomolecule in the absence of quencher, [*Q*] the concentration of RSBs, and *K*_SV _= *K*_q_*τ*_0_ is the Stern-Volmer quenching constant. The fluorescence lifetime of biopolymer is about 10^-8^ s (19).

**Figure 3:**
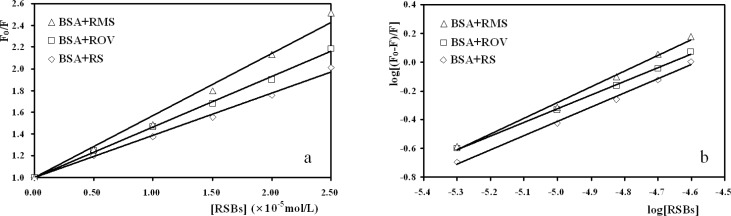
Stern-Volmer plots (a) and Double logarithm plots (b) for the interaction of RSBs with BSA ([RSBs] = 0.00, 0.50, 1.00, 1.50, 2.00, 2.50   10^-5^ mol/L, [BSA] = 1.00   10^-5^ mol/L, T = 290 K).

The Stern-Volmer plots for the interaction of the three RSBs with BSA were shown in Figure 3(a), based on the measured fluorescence data and the corresponding *K*_SV_, *K*_q_ values were calculated (Table 2). Obviously, the rate constants of BSA quenching procedure initiated by RSBs (RS, ROV, RMS) are much greater than the maximum scatter collision quenching constant of the biomolecule (*K*_q_ = 2.0 × 10^10^ L/mol·s). This means that the possible fluorescence quenching mechanism of BSA by RSBs is not initiated by dynamic collision but potentially from static quenching.

**Table 2:** Dynamic quenching constants (*K*_SV_) and quenching rate constants (*K*_q_) of RSBs+BSA solutions ([BSA] = 1.00 × 10^-5^ mol/L, T = 290 K).

	**K**_SV_ **(×** **10**^4^** L/mol)**	**K**_q_ **(×** **10**^12^ **L/mol·s)**	**R** ^2^
BSA+RS	3.883	3.883	0.9956
BSA+ROV	4.648	4.648	0.9980
BSA+RMS	5.709	5.709	0.9880

*Binding constants an* *d binding* *site* *s*

Fluorescence intensity data can also be used to obtain the binding constant (*K*_a_) and the binding site numbers (*n*). Equilibrium between free and bound molecules is given by equation (2) providing that small molecules bind independently to a set of equivalent sites on a macromolecule:

Equation (2) log(F0-F)/F= log Ka+ n log[Q]

According to the above equation, the double logarithm plots were obtained (Figure 3(b)). *K*_a_ and *n* values were calculated and listed in Table 3. The correlation coefficients are all larger than 0.99 indicating that the interaction between RSBs and BSA agrees well with the site-binding model underlined in the above equation. As can be seen in Table 3, the values of *K*_a_ are larger than 10^4^, which indicate that there is a strong interaction between the three RSBs and BSA. The order is *K*_a_ (BSA + RMS) > *K*_a_ (BSA + RS) > *K*_a_ (BSA + ROV). The values of *n *are all approximately equal to 1, indicating that there is one class of binding site for the three RSBs towards BSA. 

**Table 3:** Apparent binding constants (*K*_a_) and binding sites (*n*) of the interaction of RSBs with BSA

	**K**_a_ **(×** **10**^4^** L/mol)**	**n**	**R** ^2^
BSA+RS	3.251	0.9848	0.9954
BSA+ROV	2.749	0.9523	0.9976
BSA+RMS	16.029	1.0968	0.9932

*The binding distance between RSBs* *(RS, ROV, RMS) and BSA*

According to the Förster’s theory, the efficiency of energy transfer, *E*, is given by:

Equation (3) $$E=1－F/F_{0}=1/[1+{\left(r/R_{0}\right)}^{6}]$$

The *F*_0_ and *F* are the fluorescence intensities of BSA solutions with 1.00 × 10^-5^ mol/L at 340 nm in the absence and presence of RSBs (RS, ROV, RMS) with 1.00 × 10^-5^ mol/L, respectively. The *r* is the donor-acceptor distance and *R*_0_ is the distance at 50 % transfer efficiency. The value of *R*_0_^6^ can be calculated using the equation (4):

Equation (4) $$R_{0}^{6}=\mathrm{8.8}\times 10^{-25}(K^{2}n^{-4}\varphi_{D}J)$$

Where the *K*^2^ is the spatial orientation factor of the dipole, *n* is the refractive index of medium, *φ*_D_ is the quantum yield of the donor in the absence of acceptor and *J* is the overlap integral of the emission spectrum of the donor (BSA) and the absorption spectrum of the acceptor (RSBs). In the present case, *K*^2^, *n* and *φ*_D_ are 2/3, 1.336 and 0.15 for BSA (20), respectively. And then, the *J* can be calculated by the equation:

Equation (5) $$J=\mathrm{\Sigma F}\left(\lambda \right)\varepsilon \left(\lambda \right)\lambda^{4}∆\lambda /\mathrm{\Sigma F}\left(\lambda \right)∆\lambda$$

**Figure 4:**
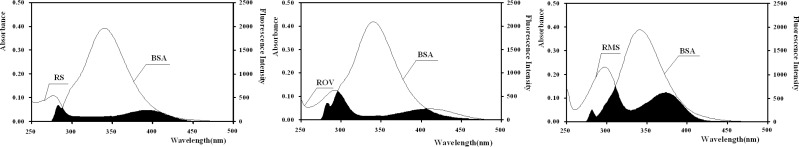
Spectral overlap of fluorescence ( _ex_ = 280 nm) of BSA solution and absorption of RSBs (RS, ROV, RMS) solutions ([BSA] = [RSBs] = 1.00   10^-5^ mol/L).

Where *F*(*λ*) is the ﬂuorescence intensity of the donor in the wavelength range λ to λ + Δλ and *ε*(*λ*) is the absorption coefﬁcient of the acceptor at *λ*. Figure 4 shows the spectral overlap of fluorescence emission of BSA solution and UV-vis absorption of the three RSBs solutions, respectively. From these relationships, *J*, *E*, *R*_0_ and* r* of the three RSBs + BSA solutions can be calculated and the corresponding results were given in Table 4. Apparently, the donor-acceptor distances are all less than 7.0 nm, which indicates that the energy transfer from BSA to RSBs (RS, ROV, RMS) occurs with high possibility. It also suggested that the bindings of these three RSBs to BSA molecules were formed through energy transfer, which quenched the fluorescence of BSA molecules.

**Table 4:** Corresponding results according to Föster’s non-radioactive energy transfer theory ([BSA] = [RSBs] = 1.00   10^-5^ mol/L).

	**E** **(%)**	**R**_0_ **(nm)**	**J** **(cm**^3^**·L/mol)**	**r (nm)**
BSA+RS	27.28	2.2004	4.108710^-15^	2.5909
BSA+ROV	31.82	2.2269	4.415210^-15^	2.5286
BSA+RMS	32.64	2.2289	4.439110^-15^	2.5150

*The displacement experiments of site probes*

BSA is composed of a single chain of 582 amino acid residues and can be divided into three homologous domains. And each domain can be subdivided into two sub-domains (A and B). Sudlow *et al*. have suggested two main distinct binding sites on BSA, site I and site II, which locate in the hydrophobic cavities of sub-domains IIA and IIIA, respectively (21). Many ligands, such as warfarin and ketoprofen, were found to bind preferentially to site I of BSA, while ibuprofen and chlorphenamine maleate showed affinity for site II (22). In order to identify the location of the RSBs binding site on BSA, the displacement experiments were carried out using the site probes ketoprofen and ibuprofen. The ratio of RSBs to BSA was kept at 5:1 in order to keep non-specific binding of probes to a minimum. The percentage of fluorescence probe displaced by the site probes was determined by measuring the changes in fluorescence intensity according to the method proposed by Sudlow *et al*. (19):

Equation (6) $$F_{2}/F_{1}\times \mathrm{100}\%$$

**Figure 5:**
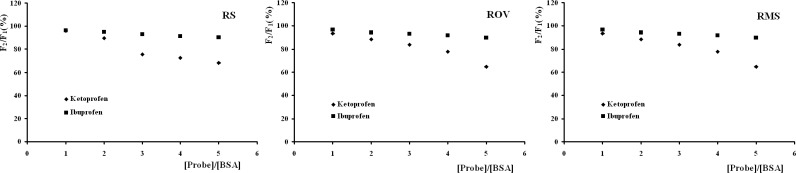
Effect of site probes on the fluorescence of RSBs+BSA solutions ([BSA] = 1.00 × 10^-5^ mol/L, [RSBs] = 5.00 × 10^-5^ mol/L, λ_ex_ = 280 nm, λ_em_ = 336 nm).

The *F*_1_ and *F*_2_ denote the fluorescence of the RSBs + BSA solutions without and with the probe, respectively. The plots of *F*_2_/*F*_1_ against [probe]/[BSA] were shown in Figure 5. It can be seen that the fluorescence intensities were all remarkably affected by adding ketoprofen, while the changes were not significant with the addition of ibuprofen. These results indicated that ketoprofen displaced RSBs from the binding site while ibuprofen had a little effect on the binding of RSBs to BSA. Hence, we can conclude that site I is the main location for the three RSBs binding to BSA. 

*Synchronous fluorescence spectra*

**Figure 6:**
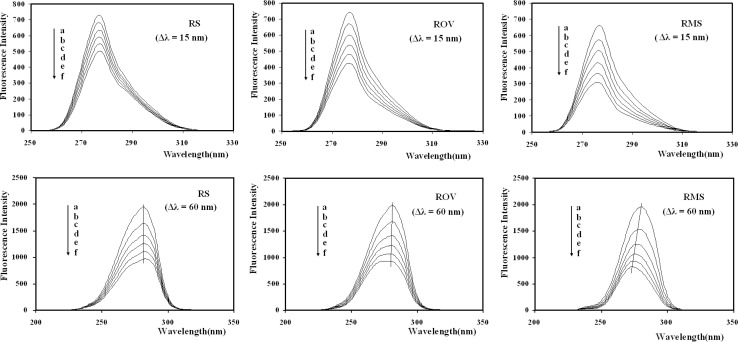
Synchronous fluorescence quenching spectra of BSA in the presence of RSBs (RS, ROV, RMS). ([RSBs] (a→f) = 0.00, 0.50, 1.00, 1.50, 2.00, 2.50   10^-5^ mol/L; [BSA] = 1.00   10^-5^ mol/L).

Synchronous fluorescence measurements provide information about the molecular microenvironment in the vicinity of the fluorophore functional groups. The maximum emission wavelengths of the residues are related to the polarity of the surrounding environment. Synchronous fluorescence spectra were obtained by simultaneously scanning excitation and emission monochromators. If Δλ = 15 nm, the synchronous fluorescence spectrum exhibits the spectral character only of tyrosine residues, and if Δλ = 60 nm, it exhibits that only of tryptophan residues. Synchronous fluorescence spectra of BSA upon addition of RSBs (RS, ROV, RMS) gained at Δλ = 15 and 60 nm were shown in Figure 6.

As shown in Figure 6, the quenching of the fluorescence intensity of all the three tryptophan residues are stronger than that of tyrosine residues, suggesting that tryptophan residues contribute greatly to the quenching of intrinsic fluorescence of BSA. Moreover, there was a slight blue shift of maximum emission wavelength of tryptophan residues with the addition of ROV or RMS, whereas no signiﬁcant shift was observed with the addition of RS. It was likely due to that the polarities around tryptophan residues were decreased, the hydrophobicity was increased and the amino acid residues were less exposed to the solvent with the addition of ROV or RMS. It also can be seen that the tyrosine residues emission maximum kept unchanged, suggesting that the polarity around tryptophan residues had no remarkable change during the binding process. This indicates that the methoxy (OCH_3_) group can change the interaction model of RSBs with BSA molecules.

## Conclusion

Three new RSBs (RS, ROV and RMS) were successfully synthesized. Interactions between these three RSBs with BSA molecules were investigated and the experimental results indicated that these three RSBs can interact with BSA strongly in sub-domain IIA mainly through the hydrophobic interaction, which change the conformation of BSA. The binding degree of these three RSBs with BSA molecules ranks in the order of RS < ROV < RMS. The protein fluorescence quenching mechanism was mainly a static quenching process and the methoxy groups on the aromatic ring of drug molecules played an important role during the binding processes. This study is expected to provide important insight into the interactions of serum proteins with RSBs.
